# Physiological Roles of Red Carrot Methanolic Extract and Vitamin E to Abrogate Cadmium-Induced Oxidative Challenge and Apoptosis in Rat Testes: Involvement of the Bax/Bcl-2 Ratio

**DOI:** 10.3390/antiox10111653

**Published:** 2021-10-21

**Authors:** Ahmed Abdel-Wahab, Kamel M. A. Hassanin, Ahmed A. Mahmoud, Walaa I. E. Abdel-Badeea, Abdel-Razik H. Abdel-Razik, Eman Zekry Attia, Usama Ramadan Abdelmohsen, Rabie L. Abdel Aziz, Agnieszka Najda, Ibtesam S. Alanazi, Khalaf F. Alsharif, Mohamed M. Abdel-Daim, Mohamed O. Mahmoud

**Affiliations:** 1 Physiology Department, Faculty of Veterinary Medicine, Minia University, Minia 61519, Egypt; 2 Biochemistry Department, Faculty of Veterinary Medicine, Minia University, Minia 61519, Egypt; kamelmagha64@mu.edu.eg (K.M.A.H.); walaa.vet@mu.edu.eg (W.I.E.A.-B.); 3 Chemistry Department, Faculty of Sciences, Minia University, Minia 61519, Egypt; amahmoud@mu.edu.eg; 4 Histology Department, Faculty of Veterinary Medicine, Beni-Suef University, Beni-Suef 62511, Egypt; abdelrazak.osman@vet.bsu.edu.eg; 5 Pharmacognosy Department, Faculty of Pharmacy, Minia University, Minia 61519, Egypt; eman_zekry@mu.edu.eg (E.Z.A.); usama.ramadan@mu.edu.eg (U.R.A.); 6 Pharmacognosy Department, Faculty of Pharmacy, Deraya University, Universities Zone, New Minia City 61111, Egypt; 7 Theriogenology Department, Faculty of Veterinary Medicine, Beni-Suef University, Beni-Suef 62512, Egypt; rabea.ramadan@vet.bsu.edu.eg; 8 Department of Vegetable Crops and Medicinal Plants, University of Life Sciences in Lublin 50A Doświadczalna Street, 20-280 Lublin, Poland; agnieszka.najda@up.lublin.pl; 9 Department of Biology, Faculty of Sciences, University of Hafr Al Batin, Hafr Al Batin 39524, Saudi Arabia; esalanazy@uhb.edu.sa; 10 Department of Clinical Laboratory Sciences, College of Applied Medical Sciences, Taif University, P.O. Box 11099, Taif 21944, Saudi Arabia; alsharif@tu.edu.sa; 11 Department of Pharmaceutical Sciences, Pharmacy Program, Batterjee Medical College, P.O. Box 6231, Jeddah 21442, Saudi Arabia; 12 Pharmacology Department, Faculty of Veterinary Medicine, Suez Canal University, Ismailia 41522, Egypt; 13 Biochemistry Department, Faculty of Pharmacy, Beni-Suef University, Beni-Suef 62514, Egypt; mohamed.omar@pharm.bsu.edu.eg

**Keywords:** red carrot methanolic extract, cadmium chloride, Vitamin E, oxidative stress, Bax/Bcl-2 ratio

## Abstract

The precise analysis of the contents of the red carrot is still ambiguous and its role in the maintenance of male fertility needs to be further reconnoitered. Hence, this study targets the physiological impacts of either red carrot methanolic extract (RCME) or vitamin E (Vit. E), co-administrated with cadmium chloride (CdCl_2_) on rat testes, specifically those concerned with apoptosis and oxidative challenge. Four groups of adult male rats (*n* = 12) are used; control, CdCl_2_, CdCl_2_ + Vit. E and CdCl_2_ + RCME. LC-MS analysis of RCME reveals the presence of 20 different phytochemical compounds. Our data clarify the deleterious effects of CdCl_2_ on testicular weights, semen quality, serum hormonal profile, oxidative markers and Bax/Bcl-2 ratio. Histopathological changes in testicular, prostatic and semen vesicle glandular tissues are also observed. Interestingly, our data clearly demonstrate that co-administration of either RCME or Vit. E with CdCl_2_ significantly succeeded in the modulation (*p* < 0.05) of all of these negative effects. The most striking is that they were potent enough to modulate the Bax/Bcl-2 ratio as well as having the ability to correct the impaired semen picture, oxidant status and hormonal profile. Thus, RCME and Vit. E could be used as effective prophylactic treatments to protect the male reproductive physiology against CdCl_2_ insult.

## 1. Introduction

Recently, the levels of male fertility exhibit marked decline; this is mainly attributed to the exposure of environmental pollutants even when this exposure occurs at low levels [1]. Environmental pollutants are numerous, but heavy metals, including cadmium (Cd), represent the one of the most serious, as they can induce remarkable oxidative stress conditions [2]. Cadmium exposure becomes a serious public health concern, as it is involved in the industrialization of various agents, including batteries, electronic instruments, glasses and ceramic, in addition to its usage in plastic pigmentation, steel covering and its agricultural purpose as a fertilizer [3]. Moreover, tobacco plants have been generally accepted to be the most serious environmental source for Cd [4]. As Cd discharges easily into soil, air and water, it can be conveyed easily to plants, which increases the risk of Cd exposure from dietary sources [5]. Rice, cereal grains, aquatic foods and vegetables are the most common food categories involved in Cd exposure [6]. All of these factors augment the occasion of Cd exposure to animals and humans from their surrounding environment and are responsible for its serious health hazards. In addition, the hazardous effects of Cd could be relied on for its very long biological half-life, which has been reported to be 20–40 years in humans and could last 25–30 years in animals and plants. This gives Cd the opportunity to accumulate in body tissues for a long time, resulting in an impairment of the activity of many organs, including testes [7,8].

Although programmed cell death (apoptosis) is an essential physiological pathway that helps the testis to eradicate the abnormal sperm cells, any fluctuation of controllable gene expression would induce a reduction in sperm concentration and motility [9]. Bcl-2 as an anti-apoptotic and Bax as a pro-apoptotic are increasingly becoming the most vital regulator proteins for apoptosis and their ratio represents the main indicator of cell death [10]. Cadmium is considered one of the most serious stressors that could disturb the expression of Bax/Bcl-2 ratio in testicular tissues; this mostly ends with the enhancing of apoptosis [11,12]. In addition, Cd could also affect male fertility by damaging Sertoli and Leydig cells as well as the testicular blood barrier [13]. Intra-peritoneal injection of rats with Cd for 21 days resulted in severe histological damage in the testicular tissue together with an alteration of sperm cell activity [14]. Moreover, Cd was reported to accumulate in serum and testes when administered for 4 weeks; this induced depletion of testicular enzymatic antioxidants, impairment of serum gonadotrophin hormones and the enhancing of testicular autophagy [15]. Furthermore, Cd decreased libido, fertility and testosterone levels [16]. In addition, Cd as an endocrine disruptor chemical impedes the release of male hormones, including testosterone and gonadotropins [17]. It has been noted that Cd could induce oxidative stress in different tissues, including testis [18]. In this observation, Cd administration provoked an abundance of oxidative markers (malondialdehyde “MDA”) in testes, along with the elimination of enzymatic and non-enzymatic antioxidants, resulting in spoiling semen quality [19].

Vitamin E (Vit. E) is considered an ideal antioxidant, as it can reach to different body tissues depending on its lipophilic activity, keeping them from oxidative stress [20]. There are some studies clarifying the antioxidant roles of Vit. E in the protection of testes from oxidative stress [21]. In this regard, the exposure of rats to radio frequency waves remarkably eliminated the enzymatic antioxidants in the testis; this was rectified significantly with Vit. E [22]. In addition, Vit. E succeeded to abrogate the oxidative effect of Cd and maintained the enzymatic antioxidant levels within a normal range [23].

Recently, plants including polyphenolic compounds were proved to be effective natural antioxidants in addition to having achieved markedly significant protection against Cd toxicity [24]. One of these plants is the carrot, which is a special vegetable related to the Apiaceae family and has been observed to contain excessive amounts of natural antioxidant compounds [25]. It has been demonstrated that the carrot is an essential source of trace elements, vitamins and antioxidant constituents, which are mainly beta carotene and polyphenolic compounds [26]. Carrot has been also used in relieving diseases, depending on its content of carotenoid and polyphenols [27]. There are many types of carrot, but the antioxidant content in the red carrot has been noticed to be higher than other types (orange and yellow), in addition to containing high levels of polyphenols [28].

The carrot has been used traditionally in different medical purposes. Despite this interest, no one, to the best of our knowledge, has studied the application of the red carrot in the defense against Cd-induced male infertility. Therefore, the current study was conducted to explore if we could take advantage of the properties of a red carrot methanolic extract (RCME) and Vit. E to safeguard against the physiological activities of male reproduction from the hazardous effects of Cd, specifically those concerned with hormonal alteration, sperm cells impairment, oxidative challenge and apoptosis. This is assisted by metabolomic profiling using the HR–LC–ESI–MS technique, in order to illustrate the phytochemical components of RCME, which might be accountable for bioactivity.

## 2. Materials and Methods

### 2.1. Chemicals

In the present work, all chemicals used were purchased from Sigma–Aldrich, St. Louis, MO, USA, unless otherwise stated.

### 2.2. Preparation of Red Carrot Methanolic Extract

The roots of the red carrot (Daucus carota L.) were obtained from the EL-Minia main vegetable and fruit market, El-Minia city, Egypt. Then, they were identified by experts in the Botany Department, Faculty of Sciences, Minia University, El-Minia, Egypt. A one-time preparation of the RCME was according to the protocol of Zykevičiūtė-Laugks et al. [29]. In short, fresh tuber roots of red carrot (1kg) were striped, washed, cut into small pieces and extracted with methanol–water (1:1) at room temperature for three days. The extract was filtered and concentrated using a vacuum rotary evaporator. The obtained red carrot residue (180 g) was aliquoted into vials, each containing one gram, and stored at −20 °C for the experimental study. The residue in each vial was then resuspended in a normal physiological saline (0.9% NaCl) to reach a final concentration of 1g/mL prior to administration. After each daily administration, the remaining residue was kept at 4 °C for use the next day.

### 2.3. Phytochemical Analysis and the Assay of In Vitro Antioxidant Activities of Red Carrot Methanolic Extract (RCME)

The red carrot methanolic extract was subjected to phytochemical analysis and the assay of in vitro antioxidant activities in the Department of Pharmacognosy, Faculty of Pharmacy, Minia University, as follows: (1) Total phenols content of RCME were analyzed following the method described by Singleton et al. [30], while estimation of the total flavonoids content was done by a modified colorimetric method, described by Benariba et al. [31]. (2) Antioxidant activities assays of RCME were assessed through determining the free radical scavenging potentials of the RCME spectrophotometrically using 1, 1-diphenyl-2-picryl-hydrazyl (DPPH), as previously described [32] and the total reducing abilities by the phosphomolybdate complex method, in which the ascorbic acid was used as a standard, was performed according to the method described by Jan et al. [33].

### 2.4. LC–MS Analysis

One milligram of dried RCME was dissolved in methanol (HPLC-grade) to access 1 mg/mL as a final concentration and then subjected to a high resolution liquid chromatography–electrospray ionization–mass spectroscopy analysis using analyzers of Thermo Scientific Exact mass (Thermo Scientific, Karlsruhe, Germany) connected to a HPLC system (Dionex UltiMate 3000). Certain requirements were applied, including: 260 °C as a capillary temperature, 45 V as a capillary voltage, a sheath gas flow rate of 40–50 arbitrary units, spray voltage 4.5 kV, an auxiliary gas flow rate of 10–20 arbitrary units and a mass range of 100–2000 amu (maximum resolution 30,000). The sample was eluted through a column (C-18, 75 mm length, 3.0 mm diameter and 5 μm particle size) (ACE, Mainz, Germany). The elution was carried out using a mobile phase comprised of 0.1% formic acid in water (HPLC-grade) (solvent A) and acetonitrile HPLC-grade (solvent B). The flow rate was adjusted at 300 µL/min. Gradient elution was initiated with 10% solvent B for 5 min, which over 30 min was increased to 100% solvent B. It was retained for 5 min at 100% solvent B and then decreased to 10% solvent B in the next minute to equilibrate the column with 10% solvent B for 4 min until the end of the run. A mass analyzer of LTQ Orbitrap Thermo scientific (Thermo Scientific, Karlsruhe, Germany) was used to measure MS/MS.

**Figure 1:**
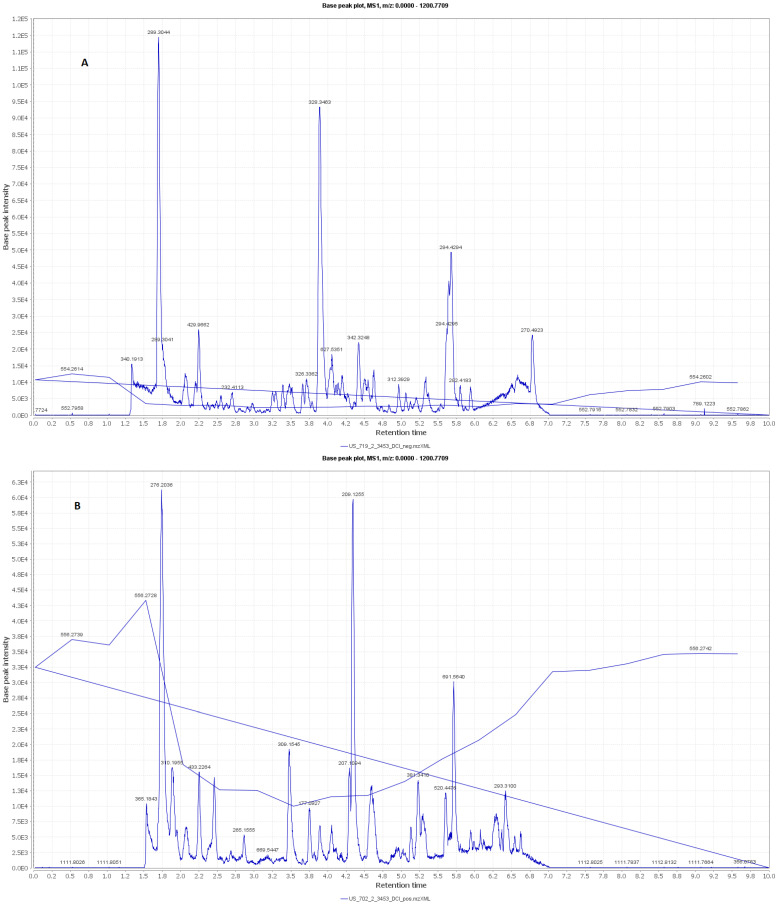
LC–MS total ion chromatogram (**A**) positive mode, (**B**) negative mode of the RCME.

In addition, metabolomic analyses were also carried out. Secondary metabolites of the RCME were tentatively identified with the assistance of current high resolution mass and by searching online and in-house databases. The extract components analysis was accessed using Xcalibur 3.0, Thermo Fisher Scientific Inc., Waltham, MA, U.S. and dereplicated using the Dictionary of Natural Products database V. 23.1 on DVD. The total ion chromatograms of positive and negative modes were shown in Figure 1.

### 2.5. Animals and Experimental Design

In the present work, all procedures applied to the rats were fulfilled following the guidelines of the local Animal Care and Use Committee of Research for the Faculty of Veterinary Medicine, Minia University, Egypt. Forty-eight adult Wistar albino male rats of average weight 167.60 ± 3.99 g BW, obtained from the lab animal center, Faculty of Veterinary Medicine, Beni-Suef University, were used in this study. Two weeks of adaptation under normal environmental condition (22 ± 3 °C and with 12 h light/12 h dark cycle) were required to begin the study. All rats were kept in plastic cages with ad libitum drinking water and feeding using normal rat diet (21% protein, 3% crude fiber, 7% fat, 1% vitamins and mineral premix, and 68% yellow corn “2490.00 Kcal/kg”).

The rats were randomly divided into four groups (*n* = 12) and all animals were gavaged daily with their corresponding treatments for 2 months. The groups included the control group (rats were gavaged corn oil as a vehicle), CdCl_2_ group (rats were gavaged 5 mg CdCl_2_/kg BW dissolved in normal physiological saline “0.9% NaCl”) as outlined by Hassanin and Safwat [34], CdCl_2_ + Vit. E group (rats were gavaged with a combination of 5 mg CdCl_2_/kg BW and 400 mg Vit. E/kg BW dissolved in corn oil) according to the method depicted by Layachi and Kechrid [35] and CdCl_2_ and RCME group (rats were gavaged with 5 mg/kg BW CdCl_2_ and 400 mg RCME/kg suspended in physiological saline) according to the method depicted by Sodimbaku et al. [36].

We gavaged the rats in the control group with corn oil (The vehicle for dissolving Vit. E), not saline (the vehicle for dissolving CdCl_2_ and RCME), as it has been established that saline has no effects on rats’ testes [17]. Consequently, we used the corn oil for control group to exclude the augmented effect caused by its use with vitamin E in Vitamin E and CdCl_2_ group. Our protocol met an agreement with a recent study of Fang et al. [37], who also used the corn oil as a vehicle for control group and not the other vehicle (distilled water).

### 2.6. Body Weight Gain, Testes Weights and Gonadosomatic Index

Body weights of rats were recorded at day zero (first day of experiment) and at the end of the experiment (on the sixtieth day), to calculate means of body weight gain. Additionally, after the rats were sacrificed on the sixtieth day, testes from each rat were collected and weighed. In addition, the average testes weights were divided by the corresponding body weights and then multiplied by 100 to record the gonadosomatic index (GSI).

### 2.7. Serum and Tissue Samples

On the sixtieth day, rats were prepared in order to bleed their eyes and deposit the blood samples in sterile dry tubes. After waiting one hour at room temperature, centrifugation was done to separate serum samples that were kept at −20 °C until the time of measuring the studied parameters.

Concerning tissue preparation, after euthanasia, the abdomen was opened. The testes, prostate gland and semen vesicle gland were collected. The testes were collected and divided into 3 parts. The first part was suspended in physiological saline (0.9% NaCl) containing RNase and protease inhibitors (Promega Corporation, 2800 Woods Hollow Road, Madison, WI 53711 USA) and then was kept at −80 °C for the measurement of the apoptotic “Bax” (gene accession number: NM_017059) and anti-apoptotic “Bcl-2” (gene accession number: NM_016993) markers by RT-qPCR. The second part (0.5 g) was homogenized in 5 mL phosphate-buffered saline (NaCl 8 g/L, KCl 0.2 g/L, Na_2_HPO_4_ 1.44 g/L and KH_2_PO_4_ 0.24 g/L) by using tissue homogenizer (Yellow line DI 18 basic, Deutschland, Germany). This was followed by the centrifugation of the testicular tissue homogenates for 15 min at 10,000 rpm. The supernatants were collected and stored at −20 °C for measurement of some oxidative stress markers. The third part of the testes and the prostate gland, as well as the semen vesicle gland, were placed in Bouin’s solution (75 mL of picric acid saturated aqueous solution, 25 mL of 40% aqueous solution of formalin and 5 mL of acetic acid glacial) for histopathological examination.

### 2.8. Epididymal Semen Samples Collection and Evaluation

The dissection of epididymis were done on clean warm glass slide with a few drops of warm distilled water added to it to collect the semen sample. Sperm count was assessed using a Neubauer chamber, as previously described [38]. In addition, semen smears were immediately prepared and stained with eosin and nigrosine to detect primary (including sperm cells with coiled tails, rudimentary tails, macroheads, microheads and double tails) and secondary sperm abnormalities (including sperm cells with curved tails, bent tails, headless tails, tailless heads and looped tails), as previously outlined [39].

### 2.9. Hormonal Assay

FSH, LH and testosterone hormones were measured using specific ELISA kits according to the manufacture’s protocol. The rodent FSH and LH ELISA kits (catalogue numbers: KA2330 and KA2332, respectively) were purchased from Abnova corporation (P.O. Box 1697, Walnut, CA 91788, USA). The rat testosterone ELISA Kit (catalogue number: OKCA00179) was obtained from Aviva Systems Biology, Corp. (7700 Ronson Road, Ste 100 San Diego, CA 92111, USA).

### 2.10. Determination of Oxidative Stress Markers

All studied oxidative stress markers were estimated in testicular tissue homogenates using specific colorimetric assay kits (Sigma–Aldrich, St. Louis, MI, USA) according to the manufacturer’s instruction. The measuring of the malondialdehyde (MDA) concentration depends on a reaction done in acidic medium for 30 min between thiobarbituric acid (TBA) and MDA at 95 °C. This reaction forms a thiobarbituric acid reactive product. At 534 nm, the absorbance of the resulted colored substance was done. Concerning nitric oxide concentration (NO), this test also needed an acidic medium in addition to the presence of nitrite. In this case, the obtained nitrous acid diazotized sulphanilamide and the product is coupled with N-(1-naphthyl) ethylenediamine (NEDA). At 540 nm, the absorbance of the produced azo dye of reddish color was determined. For measuring catalase (CAT), it firstly reacts with H_2_O_2_ (known amount), then after one minute, the reaction was stopped using the catalase inhibitor. The residual H_2_O_2_ reacts with 3,5-dichloro-2-hydroxybenzene sulfonic acid (DHBS) and 4-aminophenazone (AAP) in the presence of peroxidase (HRP), to form a colored material that is inversely proportional to the amount of catalase in the original sample. The principle of superoxide dismutase (SOD) assay mainly relies on an inhibitory reaction catalyzed by SOD for the phenazine methosulphate-mediated reduction of nitroblue tetrazolium dye (NBT). The reduced glutathione (GSH) test is mainly based on the ability of GSH to reduce 5,5′-dithiobis-2-nitrobenzoic acid (DTNB), which resulted in a yellow colored product. At 405 nm, the absorbance of this colored material was measured, which is directly proportionate to the GSH concentration in the sample.

### 2.11. Determination of mRNA Relative Expression Levels of Bcl-2 and Bax Genes in Testicular Tissues by Real Time Quantitative PCR (RT-qPCR):

Real time quantitative polymerase chain reaction (RT-qPCR) differs from regular PCR by including the reaction fluorescent reporter molecules that increase proportionally with the increase of DNA amplification in the thermocycler. A definitive workflow of qPCR for gene expression quantification involves testicular RNA isolation, which was done by a total RNA purification kit (Jena Bioscience, Jena, Germany). Both reverse transcriptions of testicular mRNA of Bax, Bcl-2 and β-actin genes and their qPCR assay were conducted in one step assay by using the GoTaqR 1-Step RT-qPCR System kit (Promega Corporation, 2800 Woods Hollow Road, Madison, WI 53711, USA). The relative quantification of the mRNA expression of testicular Bax and Bcl-2 was calculated according to the Applied Biosystem Software included in the Applied Biosystems Real-Time PCR Instruments (Thermo fisher scientific, Waltham, MA, USA). The primers used for Bax, Bcl-2 and β-actin (gene accession number: NM_007393) (as internal control) were (Forward primer: 5′-GGGGACGAACTGGACAGTAACAT-3′ and reverse primer 5′-GGAGTCTCACCCAACCACCCT-3′ for Bax), (Forward primer: 5′-CATGTGTGTGGAGAGCGTCAA-3′ and reverse primer 5′-GCCGGTTCAGGTACTCAGTCA-3′ for Bcl-2) and (Forward primer: 5′- ATGAGCCCCAGCCTTCTCCAT-3′ and reverse primer 5′- CCAGCCGAGCCACATCGCTC-3′ for β-actin). The thermal cycler program used was as follows: One cycle for reverse transcription at 37 °C for 15 min and one cycle of RT inactivation/hot-start activation at 95 °C for 10 min, then 40 cycles of qPCR (10 s for denaturation at 95 °C, 30 s for annealing at 60 °C and 30 s for extension at 72 °C), followed by a final extension (one cycle) at 72 °C for 10 min.

### 2.12. Histopathological Examination

The histopathological examination was done in the histopathology laboratory of the Department of Histology, Faculty of Veterinary Medicine, Beni-Suef University, as previously outlined [40]. The first step was the fixation of testicular, prostatic and semen vesicle tissues samples using Bouin’s solution. This step was followed by dehydration using ethanol with graded concentrations and then embedded in paraffin and sectioned at 2–5 µm thickness. This was followed by staining with hematoxylin–eosin (H & E) and periodic acid–Schiff (PAS) as a special stain for glandular secretion. Finally, these stained films were examined with a LEICA light microscope with 10× and 20× objective lens.

### 2.13. Statistical Analysis

In this study, all data were applied for statistical analyses using IBM SPSS statistics 20 software. The test used was a one-way ANOVA test, followed by the Tukey post-hoc test for multiple comparisons. All values were stated as means ± standard error of mean (SEM). The significant differences for the output data were considered when *p* < 0.05.

## 3. Results

### 3.1. Phytochemical Analysis and Assay of In Vitro Antioxidant Activities of the Red Carrot Methanolic Extract

The results of the phytochemical analysis and in vitro antioxidant activities of the RCME revealed that the total phenolic content was 38.46 ± 5.2 mg/g dry RCME expressed as a gallic acid equivalent, and the total flavonoid content was 22.32 ± 2.8 mg of quercetin equivalents (QE)/g dry RCME. Moreover, RCME showed substantial antioxidant activity with an efficient concentration 50 (EC50) value of 32.79 mg/mL, using DPPH scavenging activity, and 16.19 ± 0.40 mg ascorbic equivalent/g dry extract on the phosphomolybedate complex assay.

### 3.2. LC–MS Metabolomic Analysis of the Red Carrot Methanolic Extract

**Figure 2:**
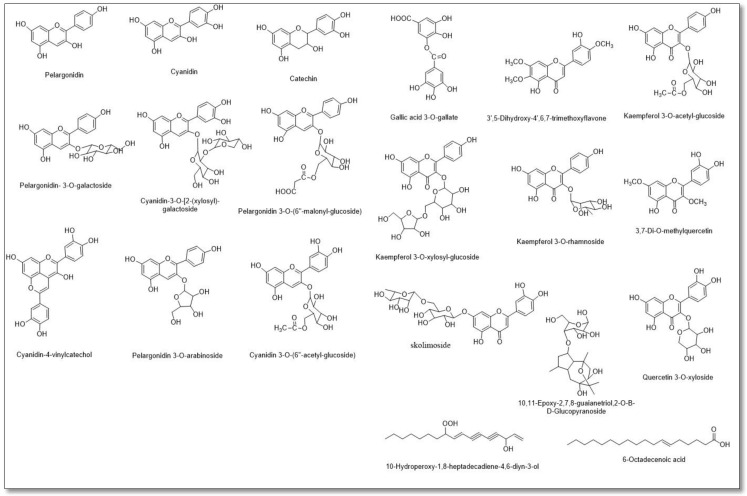
Formula of the compounds resulting from LC–MS metabolomic analysis of the red carrot methanolic extract.

A total of 20 compounds were characterized from the HR–LC–ESI–MS analysis of the RCME (Table 1 and Figure 1 and Figure 2), with a noticeable abundance of anthocyanins that were principally dominated by cyanidin, pelargonidin and their derivatives. Moreover, a number of flavonols, mainly kaempferol and quercetin derivatives, along with flavones, namely 3′,5-Dihydroxy-4′,6,7-trimethoxyflavone and skolimoside, flavanol (catechin) and polyphenolic acid (gallic acid 3-O-gallate), were also identified herein. Besides the characterized phenolic components, a number of other metabolites related to different classes were also detected, including, sesquiterpene (10,11-Epoxy-2,7,8-guaianetriol,2-O-β-D-glucopyranoside), fatty alcohol (10-hydroperoxy-1,8-heptadecadiene-4,6-diyn-3-ol) and acid (6-octadecenoic acid).

### 3.3. Means of Body Weight Gain, Testes Weights and Gonadosomatic Index in Control and Treated Rats

**Figure 3:**
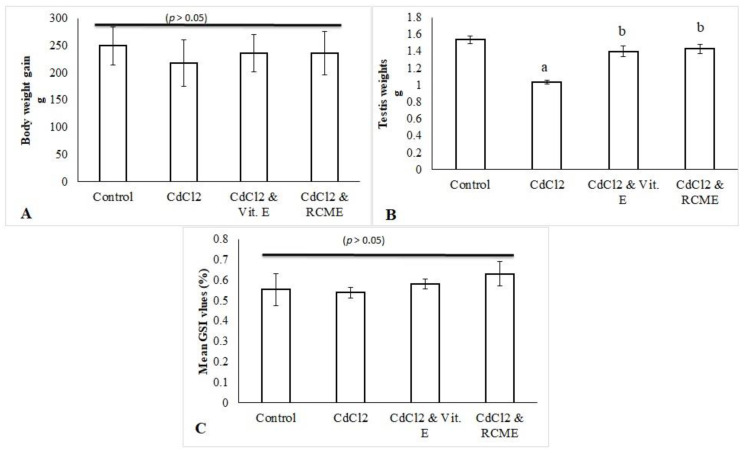
Mean values in grams of body weight gain (**A**) and testes weights (**B**) as well as the mean values of GSI (**C**) in control and treated groups. Each bar represents the mean ± SEM. a. *p* < 0.05 versus normal control. b. *p* < 0.05 versus CdCl_2_ group. RCME: Red carrot methanolic extract; Vit. E: Vitamin E; GSI: gonadosomatic index.

As shown in Figure 3A, mean values of the body weight gain of CdCl_2_-treated rats were comparable (*p* > 0.05) to other groups. Concerning testicular weights, the mean values were reduced significantly with CdCl_2_ (*p* < 0.05) while they were upturned significantly toward control level (*p* < 0.05) by the co-administration of CdCl_2_ with either RCME or Vit. E (Figure 3B). However, the GSI mean values were observed to be consistent among all groups (*p* > 0.05).

### 3.4. The Status of Semen Quality in Control and Treated Rats

**Figure 4:**
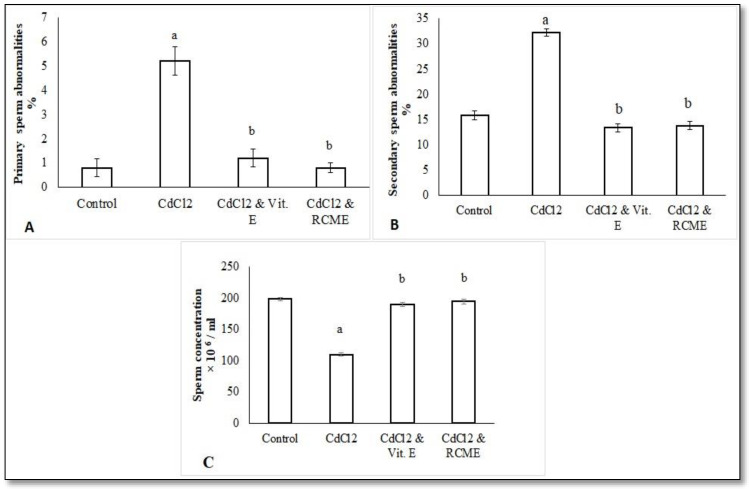
Mean values of primary (**A**) and secondary (**B**) sperm abnormalities as well as sperm concentration (**C**) in epididymal semen samples of control and treated rats. Each bar represents the mean ± SEM. a, *p* < 0.05 versus normal control. b. *p* < 0.05 versus CdCl_2_ group. RCME: Red carrot methanolic extract.Vit. E: Vitamin E.

All data about semen quality in control and treated rats were displayed in Figure 4. The observed primary sperm abnormalities (including sperm cells with coiled tails and rudimentary tails) and secondary ones (including sperm cells with curved tails, bent tails, headless tails, tailless heads and looped tails) were significantly increased with CdCl_2_ (Figure 4A,B), while they were significantly improved with the co-administration of CdCl_2_ with either Vit. E or RCME, in comparison to the CdCl_2_ group (*p* < 0.05). Moreover, CdCl_2_ administration reduced sperm concentrations markedly (Figure 4C) while Vit. E or RCME co-administration with CdCl_2_ exhibited significant enhancement when compared with the CdCl_2_ group (*p* < 0.05).

### 3.5. Serum Levels of Male Reproductive Hormones (FSH, LH and Testosterone) in Control and Treated Rats

As shown in Table 2, compared to the control group, the CdCl_2_ treatment significantly increased the serum levels of FSH whilst those of both LH and testosterone were significantly reduced (*p* < 0.05). Interestingly, the co-administration of either Vit. E or RCME with the CdCl_2_ induced significant increase in LH and testosterone concentrations as well as significant modulation of the elevated FSH concentration to control levels (*p* < 0.05).

### 3.6. Testicular Oxidant/Antioxidant Status in Control and Treated Rats

The findings of Table 2 also demonstrated the concentrations of oxidant/antioxidant markers in testicular tissue homogenate. The levels of oxidant markers (MDA and NO) were significantly elevated (*p* < 0.05) with the CdCl_2_ treatment. However, these elevated levels were significantly reduced with the co-administration of CdCl_2_ with either Vit. E or RCME when compared to the CdCl_2_ group (*p* < 0.05). In addition, the administration of CdCl_2_ significantly decreased (*p* < 0.05) the activities of testicular antioxidants (SOD, CAT and GSH). Amazingly, the co-administration of either Vit. E or RCME with CdCl_2_ significantly succeeded in modulating (*p* < 0.05) the levels of all studied antioxidants in comparison to the CdCl_2_ group and kept them within the physiological zone.

### 3.7. The Testicular mRNA Relative Expression Levels of Bax and Bcl-2 in Control and Treated Rats

**Figure 5:**
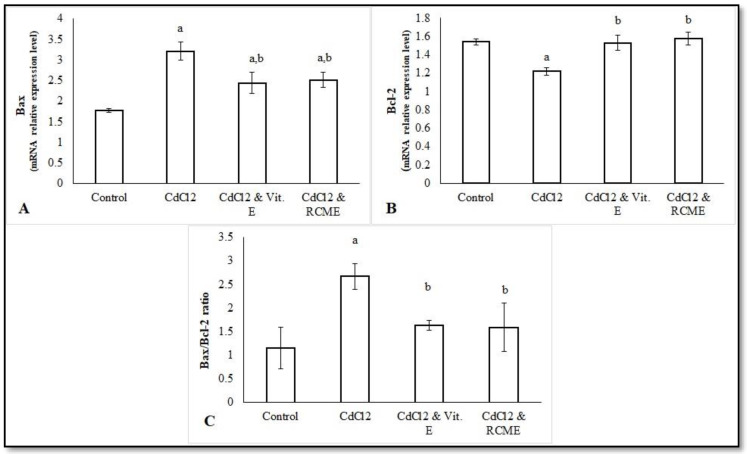
Mean values of the testicular mRNA relative expression levels of Bax (**A**), Bcl-2 (**B**) and Bax/Bcl-2 ratio (**C**) of control and treated rats. Each bar represents the mean ± SEM. a. *p* < 0.05 versus normal control. b. *p* < 0.05 versus CdCl_2_ group. RCME: Red carrot methanolic extract; Vit. E: Vitamin E; Bcl-2, B-cell lymphoma 2; BAX, Bcl-2-associated X protein.

As displayed in Figure 5, the CdCl_2_ treatment significantly impaired the Bax/Bcl-2 ratio compared to the control group (*p* < 0.05), as the mRNA relative expression levels of Bcl-2 in testicular tissues were significantly decreased, while those of Bax were significantly increased (*p* < 0.05). The most striking result is that the Bax/ Bcl-2 ratio was amended significantly (*p* < 0.05) when the rats were co-administered CdCl_2_ with either Vit. E or RCME, in comparison to the CdCl_2_ group. In this respect, the mRNA relative expression levels of Bcl-2 were significantly upturned (*p* < 0.05) and those of Bax were markedly downregulated.

### 3.8. Histopathological Findings in Testicular, Prostatic and Semen Vesicle Glandular Structures in Control and Treated Rats

**Figure 6:**
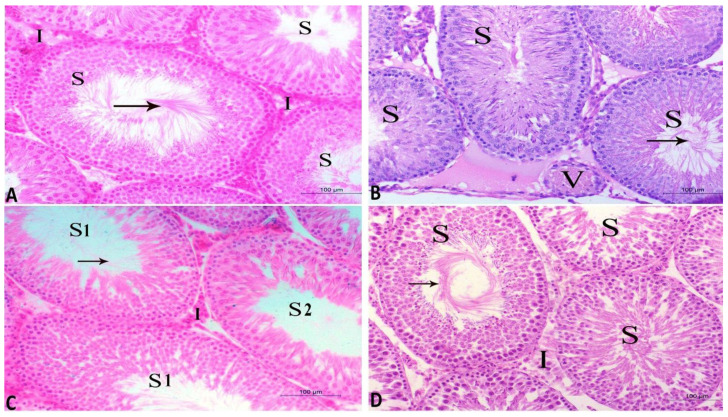
Histopathological characteristics of seminiferous tubules of testes from the four studied rat groups (**A**–**D**) using hematoxylin and eosin (H and E) staining and magnification ×200. (**A**) Control group appearance with normal seminiferous tubules (S) containing normal spermatogenic cells and spermatid as well as normal interstitial tissues (I) (**B**) CdCl_2_ group showing seminiferous tubules (S) lined with degenerated spermatogenic cells and low amounts of spermatid and sperms (arrow). Note, the interstitial tissues contain congested blood vessels (V) and inactive Leydig cells. (**C**) CdCl_2_ and Vit. E group show normal seminiferous tubules (S1). The other tubules (S2) are lined with degenerated spermatogenic cells with a few sperms. The interstitial tissues contain normal blood capillaries and less active Leydig cells (I). (**D**) CdCl_2_ and RCME group show normal seminiferous tubules (S) with spermatogenic cells and sperms (arrow). The interstitial tissues (I) appear normal.

**Figure 7:**
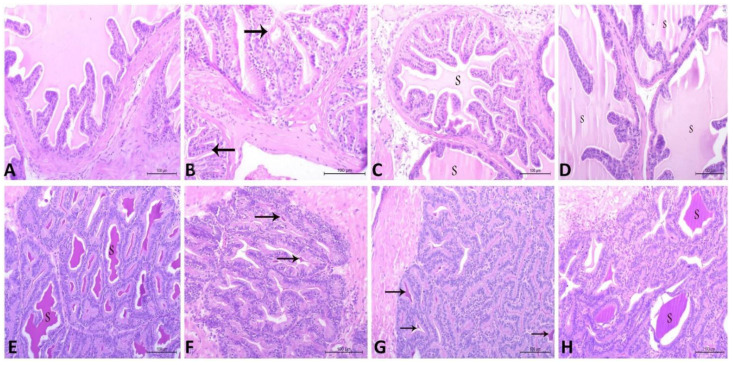
Histopathological characteristics of prostatic tissue (**A**–**D**) and vesicular gland (**E**–**H**) in adult male albino rats using hematoxylin and eosin (H and E) staining and magnification ×200. (**A**) Control group containing normal prostatic acini, lined with simple columnar secretory epithelium with secretory materials. (**B**) CdCl_2_ group showing prostatic acini, which appear collapsed and inactive and are separated by thick connective tissue. The acini are lined with low columnar epithelium with low secretory activity. Note, few secretory materials appear in the lumen of the prostatic acini (arrow). (**C**) CdCl_2_ and Vit. E group show less active prostatic acini lined with less active secretory cells and few amounts of secretory materials (S). (**D**) CdCl_2_ and RCME group show normal prostatic acini lined with simple columnar secretory epithelium, with secretory materials (S). (**E**) Control group contains normal vesicular gland acini lined with high columnar epithelium, with huge amounts of secretory materials (S). (**F**) CdCl_2_ group show vesicular gland acini appearing inactive and lined with low columnar epithelium, with few secretory materials (arrow). (**G**) CdCl_2_ and Vit. E group show active vesicular gland acini lined with active secretory cells, containing few secretory materials (arrow). (**H**) CdCl_2_ and RCME group show normal vesicular gland acini lined with simple columnar secretory epithelium and filled with secretory materials (S).

**Figure 8:**
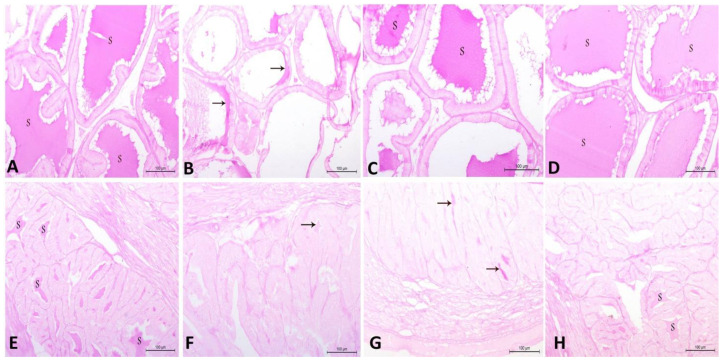
Histopathological characteristics of prostatic tissue (**A**–**D**) and vesicular glands (**E**–**H**) in adult male albino rats using the periodic acid–Schiff (PAS) stain and magnification ×200. (**A**) Control group shows prostatic tissue with strong reaction in secretory epithelium and secretory materials (S). (**B**) CdCl_2_ group shows very weak reaction in secretory epithelium and moderate reaction in secretory materials. Note, few secretory materials appear in the lumen of the prostatic acini (arrow). (**C**) CdCl_2_ and Vit. E group show strong reaction in secretory epithelium and secretory materials (S) in the majority of prostatic acini, while the others show very weak reaction in secretory epithelium and moderate reaction in secretory materials. (**D**) CdCl_2_ and RCME group show strong reaction in secretory epithelium and secretory materials (S). (**E**) Control group shows vesicular gland acini with moderate reaction in secretory epithelium and strong reaction in the secretory materials (S). (**F**) CdCl_2_ group shows very weak reaction in secretory epithelium and moderate reaction in secretory materials. Note, few secretory materials appear in the lumen of the vesicular gland acini (arrow). (**G**) CdCl_2_ and Vit. E group show moderate reaction in secretory epithelium and strong reaction in the secretory materials (arrow) in the majority of vesicular gland acini, while the others show very weak reaction in secretory epithelium and moderate reaction in secretory materials. (**H**) CdCl_2_ and RCME show vesicular gland acini with moderate reaction in the secretory epithelium and strong reaction in the secretory materials (S).

The current findings illustrate that the CdCl_2_ induced marked deteriorative changes in testicular, prostatic and semen vesicle glandular tissues. The seminiferous tubules were observed to be lined with degenerated spermatogenic cells and the lumen included a few amounts of spermatid and sperms. In addition, the interstitial area included congested blood vessels and inactive Leydig cells (Figure 6B). Furthermore, prostatic acini appeared collapsed and inactive, and were separated by thick connective tissue and lined with low columnar epithelium with low secretory activity (Figure 7B). Additionally, the secretory epithelium exhibited a faint periodic acid–Schiff (PAS) reaction while it was moderate in secretory materials (Figure 8B). Moreover, vesicular gland acini appeared collapsed and inactive and they were lined with low columnar epithelium with low secretory activity (Figure 7F). In addition, the PAS reaction was also weak in secretory epithelium and moderate in secretory materials (Figure 8F). Intriguingly, all of these degenerative changes induced by CdCl_2_ were noticeably ameliorated with both RCME and Vit. E co-administration. These histopathological improvements in testicular tissue were clearly seen (Figure 6C,D) in the form of restoring the histological picture of normal spermatogenic cells, increasing the amount of sperm cells in the tubules as well as enhancing the activity of the interstitial cells. Concerning the prostatic (Figure 7C,D and Figure 8C,D) and semen vesicle glandular tissues (Figure 7G,H and Figure 8G,H), the ameliorative effects appeared as expanded acini, lined with columnar secretory cells, which were found in deferent stages of secretory activity. The lumen of acini were observed to be obliterated with huge amount of secretory materials as well as a disappearance of thick connective tissue between the acini.

## 4. Discussion

Recently, many literatures have reported a marked decline in male fertility throughout the world; this motivated scientists to address the possible causes. Exposure to environmental pollutants was suggested to be the most contributing factor. Among all environmental pollutants, Cd represented the principle one in the induction of male infertility, as it could travel to all body organs and accumulate in high concentrations in the testes [48]. The red carrot was anciently used by Egyptians in folk medicine in different medical purposes that relied on its active biological constituents, particularly flavonoids [49]. Little is known about the role of the red carrot in improving male fertility, especially during Cd exposure, therefore, the current study was designed to investigate whether we could benefit from the properties of the active constituents of RCME that mitigate the Cd effects on the reproductive performance of adult male rats. Additionally, Vit. E was used as a standard treatment to measure the potency of RCME.

Findings of both the phytochemical and in vitro antioxidant assays along with the metabolomic analysis of RCME divulged its abundance in phenolics, including anthocyanins and flavonoids with well-known and powerful antioxidant activities. These data were consistent with previous LC-MS results for black carrot extract [50]. The anthocyanin derivative compounds of cyanidin and pelargonidin not only gave the carrot its red coloration, they also showed potent antioxidant activities [51]. Claudio and coworkers declared that the administration of purple carrot extract rich in anthocyanins ameliorated hepatic tissue degeneration and genetic injury in blood and hepatocytes promoted by cadmium intoxication [52]. Generally, it alleviated tissue degeneration, oxidative stress and genotoxicity in various organs of Wistar rats. Gallic acid 3-O-gallate and quercetin, which were enrolled in the current LC-MS analysis, are effective free radical scavengers as well [53].

The negative effects of Cd on male fertility is mainly because of the provoked oxidative stress condition [54]. About 30–80% of infertility in males is primarily due to the resulted reactive oxygen species (ROS) [55]. Malondialdehyde together with NO have been accepted to be the principal markers of oxidative and nitrosative stress in testes [56]. In the current study, CdCl_2_ administration significantly increased MDA and NO along with reducing all the studied antioxidant enzymes (*p* < 0.05). These results were consistent with previous findings [57]. Interestingly, co-administration of either Vit. E or RCME with CdCl_2_ significantly succeeded (*p* < 0.05) in reversing the negative effects of Cd. These results were in line with previous data that reported ameliorative effects for Vit. E against Cd and reinstated the normal oxidative status in testicular tissues [23]. Concerning the ameliorative effects of RCME, it mainly accounted for its active constituents, which have an efficient antioxidant power. The current LC-MS metabolomic analysis clarified the presence of quercetin and according to our phytochemical analysis, each gram of dried RCME included 22.32 ± 2.8 mg quercetin equivalents. A study of Samadder et al. [58] reported that Quercetin was effective in alleviating the oxidative stress prompted by Cd in rat testes. Additionally, Cyanidin-3-O-glucoside (one of the active compounds of RCME) when added to the diet of mice significantly enhanced the activity of SOD and GSH, along with reducing the end product of lipid peroxidation that was impaired with Cd exposure [59]. Cyanidin was considered an interested antioxidant by inhibiting the activities of xanthine oxidase enzyme; a type of enzyme that generates reactive oxygen species [60]. In addition, the phytochemical pelargonidin was observed to reverse the oxidative stress condition provoked by citrinin in vitro [61]. Moreover, depletion of MDA and abundance of GPx and GST activities in testes were accompanied with catechin treatment [62]. Thus, the protective effects of RCME against Cd-induced oxidative balance alteration mainly accounted for the activities of its constituents, primarily quercetin, cyanidin, pelargonidin and catechin, as observed in the current HR–LC–MS analysis.

In the current study, the changes of body weight caused by CdCl_2_ were statistically insignificant among all groups. This was consistent with previous results [63]. However, testes weights, but not GSI, were significantly reduced with CdCl_2_ treatment *(p <* 0.05). These results were in harmony with previously addressed findings [64]. This reduction could be attributed to the negative actions of Cd in the induction of testicular atrophy [65] and due to the impairment of the blood–testis barrier [66]. However, Vit. E and RCME co-administration with CdCl_2_ significantly ameliorated the testes weights reduction. This was supported with the reports of Kini et al. [67], who found that Vit. E maintained testes weight through the protection of its tissue from the oxidative damage induced by Cd. Concerning the roles of RCME, they are primarily explained by the actions of cyanidin and quercetin. In this regard, testicular damage induced by 3-Chloro-1,2-propanediol has been alleviated by low doses of Cyanidin-3-O-glucoside [59]. In addition, quercetin succeeded in protecting the reduction of testicular weights induced by Cd through the modulation of oxidant status [68].

Our data showed significant deterioration of semen quality with CdCl_2_ and was noticeably improved with either Vit. E or RCME. This is in consonance with previous studies that demonstrate negative influences of CdCl_2_ on sperm morphology and concentration [69]. We hypothesized that our findings, including the peroxidation of the lipid layer of spermatozoon and depletion of testicular antioxidants, together with the decline of serum levels of LH and testosterone hormones, represented the most striking reasons for the impairment of semen quality after CdCl_2_ exposure. In this regard, the generated ROS could react with the lipid layer of Leydig cells and reduce the testosterone secretion, which is essential for normal spermatogenesis [70]. This is also because the membrane of spermatozoon is rich in lipids, which is a favorable substrate for ROS to induce lipid peroxidation and increase the opportunity for sperm cell degeneration with Cd [71].

Interestingly, the co-administration of either Vit. E or RCME, alongside CdCl_2_, significantly improved the semen quality corrupted by CdCl_2_. This could be explained by our findings, which indicate that RCME does well in the elimination of MDA from the testes and enriches it with optimal levels of enzymatic antioxidant, in addition to modulating the serum levels of FSH, LH and the testosterone hormones that were impaired with CdCl_2_. These findings come in accordance with a previous study that demonstrated the protective actions of Vit. E on sperm morphology and concentration against Cd damage via the modulation of oxidant status [72]. This could be also explained by a previous study that demonstrated the essential roles for Vit. E in the protection of DNA and chromatin materials of spermatozoon from damage, along with the increasing serum levels of LH hormone [73]. Regarding RCME, it was found that the carrot improved the semen quality of men with oligospermia via increasing the catalase level in semen, which agreed with the current findings [74]. In addition, Cyanidin-3-*O*-glucoside was found to be a good candidate for improving spermatogenesis via modulating specific regulating genes that are mandatory for the normal activity of Sertoli cells, including p-ERK, p-JNK and p53 [75]. Additionally, when catechin was added in vitro to a boar semen extender, both the motility and viability of the sperms were greatly improved [76]. In vitro supplementation of semen with quercetin significantly mitigated the action of Cd; it also improved the sperm motility and viability, and kept the morphology of the membrane and promoted mitochondrial activity [77]. Therefore, the aforementioned constituents of RCME could potentiate its action to reverse the negative effects of Cd on semen quality.

The effect of Cd on the hormonal profile showed significant reduction in the serum levels of both LH and testosterone hormones and the significant elevation of FSH. Even though these results differ from some previous findings, which showed significant reduction of all hormones, even serum levels of FSH after Cd administration [78], it was found that these findings were similar to some other studies [79]. The reduction of the testosterone hormone with Cd is mostly due to its interference with the testicular levels of the LH receptors, cAMP and steroidogenic acute regulatory protein (StAR), which are mandatory for testosterone production [80]. Additionally, Cd was observed to inhibit the enzymes necessitated for testosterone production, primarily 3ß-hydroxysteroid dehydrogenase (3ß-HSD) or 17ß-HSD enzymes [81]. On a physiological basis, decreased serum testosterone levels possibly induce an increase in the levels of both FSH and LH hormones via the feedback mechanism; however, in the current study, it was found that the serum levels of LH were decreased but those of FSH were increased. This could be explained by a study of Wu et al. [79], who found that Cd induced an increase of GnRHR and FSHB expression but decreased that of LHB in pituitary cells. Another possible explanation for increasing FSH levels with Cd is due to its damaging effect on the Sertoli cells that reduce inhibin hormone production, which is the main suppressor factor on FSH [82].

As outlined in the current study, the administration of Vit. E resulted in a significant increase in serum LH and testosterone concentrations, in addition to a significant modulation of the elevated FSH levels that were induced with CdCl_2_ administration. These findings were in agreement with the study of Huang et al. [18], who reported that the expression of FSH and LH hormones in the pituitary gland were increased significantly with Vit. E. Increased gonadotropins will accordingly stimulate the Leydig cell to produce the testosterone hormone that confirms the current results.

Excitingly, the co-administration of RCME with CdCl_2_ was observed to switch the serum levels of testosterone, known as the LH and FSH hormones, from a deviation to normal state. These results are similar to a previous study that documented an increment in serum LH, which is the modulator of testosterone production, following carrot seed extract administration [83]. Recently, Cyanidin-3-O-glucoside reversed the negative effects of Cd on serum gonadotropins and testosterone, and kept their levels near control one through the modulation of both LHR and FSHR expression levels [84]. In addition, it has been observed that quercetin at 20 mg/kg BW for 28 days could reverse cadmium-induced testicular 3 as well as 17-β-HSD depletion, which are mandatory for testosterone production [85]. Furthermore, catechin is involved in steroidogenesis via increasing the activity of cAMP [86]. According to our findings, such protective effects may also be attributable to its ability to keep the normal balance between oxidants and antioxidants in the testes. Elevation of testicular antioxidants protect the testicular tissues from oxidative damage and subsequently keep the level of male sex hormones within physiological range.

When the cells are stimulated to induce apoptosis, mitochondria release the cytochrome *c* that stimulates certain caspases to induce apoptosis. These caspases have been observed to be enhanced by Bax and inhibited with Bcl-2 [87]. In the current study, CdCl_2_ significantly impaired the Bax/Bcl-2 ratio. These findings were in agreement with previous reports [88,89]. Another possible explanation for the apoptotic effects of CdCl_2_ is the increasing of NO levels, which could elicit certain apoptotic signaling pathways [90]. Interestingly, Vit. E and RCME were potent enough to ameliorate the apoptotic effects of CdCl_2_. In line with our findings, a study of Amanpour et al. [11] reported that Vit. E modulated the Cd-induced apoptosis by decreasing Bax/Bcl-2 ratio. In addition, the anti-apoptotic effect of RCME is mainly relied to its phytochemical contents. In this regard, cyanidin was effective in switching the apoptotic pathway induced by cisplatin to the normal state through the blockage of the p53 pathway. Moreover, Cyanidin-3-O-glucoside significantly succeeded in mitigating the apoptotic effect of Cd through the modulation of the MAPK signaling pathway [11]. In addition, our findings clarified that the ability of both Vit. E and RCME in defense against CdCl2-induced testicular apoptosis might be attributed to their antioxidant properties, as they significantly reduced the levels of testicular NO, which has been evidenced to be involved in the apoptotic pathway [90].

The current findings illustrate that CdCl2 induced multiple degenerative changes in the architecture of seminiferous tubules, prostatic tissues and semen vesicular glands; these changes were observed to be remarkably ameliorated with Vit. E and RCME. These findings were in agreement with previous results that observed the positive effects of Vit. E in reversing the Cd-induced histopathological changes in the rat testes [91]. The potent antioxidant action of Vit. E provides a logical explanation for such protective effects [23]. In addition, the successful action of RCME in restoring the normal architecture of the testes, prostate and semen vesicular glands mainly relies on the potent antioxidant properties of its biological active compounds (quercetin, cyanidin, pelargonidin and catechin) [59,61,62]. Therefore, the present histopathological findings confirm and fully translate our findings on the beneficial roles of RCME and Vit. E in improving testicular oxidant–antioxidant markers, semen picture and serum hormonal profile, that is deviated with Cd.

In summary, the beneficial roles of RCME that are reported in the current study against Cd-induced testicular toxicity mainly rely on the biological active phytochemical compounds that are observed by the current HR–LC–MS analysis. Our findings are augmented by the study of Li et al. [92], who reported that flavonoids, such as anthocyanins and flavonols, which are commonly found in plant foods (such as the red carrot [93]), have been observed to have protective effects against Cd-induced damage. They revealed that the cytoprotective effects of flavonoids against Cd-induced diseases are mainly attributable to three mechanisms. First, flavonoids eliminate the reactive oxygen species together with improving the activity of enzymatic antioxidants. Second, flavonoids stand against Cd accumulation in tissues through their ability of chelating Cd. Third, flavonoids alleviate DNA damage and frustrate apoptosis. It was reported that carrot consumption resulted in an elevation of plasma carrot anthocyanins after 30 min and the peak levels were reported after carrot consumption by 1.5 and 2.5 hrs [94]. Moreover, drinking carrot juice was observed to elevate the plasma antioxidant levels and reduce those of MDA [95].

## 5. Conclusions

In conclusion, RCME, relying on its valuable biological active ingredients, is observed to be an effective candidate in the protection of the testes against the hazardous effects of CdCl2 exposure. The antioxidant power of RCME is interestingly found to be comparable with that of Vit. E, one of the most ideal free radical scavengers. Both treatments significantly improved the disturbed antioxidant status, semen picture, hormonal profile and Bax/Bcl-2 ratio. They succeeded with a high degree to restore the male reproductive parameters to their approximate physiological levels.

## Figures and Tables

**Table 1:** Annotated compounds by LC–MS metabolomic analysis of the red carrot methanolic extract.

Polarity	m/z	Rt	Formula	Name	Source
[M − H]^−^	270.4925	6.8127	C_15_H_11_O_5_	Pelargonidin	[41]
[M + H]^+^	288.1700	2.103	C_15_H_11_O_6_	Cyanidin	[41,42,43]
[M + H]^+^	291.1863	6.100	C_15_H_14_O_6_	Catechin	[41]
[M + H]^+^	434.2948	3.018	C_21_H_21_O_10_	Pelargonidin- 3-O-galactoside	[42]
[M − H]^−^	580.6404	9.84	C_26_H_29_O_15_	Cyanidin-3-O-[2-(xylosyl)-galactoside	[43]
[M + H]^+^	520.4487	5.719	C_24_H_23_O_13_	Pelargonidin 3-O-(6″-malonyl-glucoside)	[42]
[M − H]^−^	490.858	4.571	C_23_H_23_O_12_	Cyanidin 3-O-(6″-acetylglucoside)	[42]
[M + H]^+^	404.2404	2.299	C_20_H_19_O_9_	Pelargonidin 3-O-arabinoside	[42]
[M + H]^+^	420.2739	2.734	C_23_H_15_O_8_	Cyanidin-4-vinylcatechol	[42]
[M − H]^−^	323.1974	4.671	C_14_H_10_O_9_	Gallic acid 3-O-gallate	[28]
[M − H]^−^	343.3254	4.604	C_18_H_16_O_7_	3′,5-Dihydroxy-4′,6,7-trimethoxyflavone	[44]
[M + H]^+^	433.2267	2.474	C_21_H_20_O_10_	Kaempferol 3-O-rhamnoside	[44]
[M − H]^−^	489.8591	4.429	C_23_H_22_O_12_	Kaempferol 3-O-acetyl-glucoside	[44]
[M − H]^−^	579.6392	1.679	C_26_H_28_O_15_	Kaempferol 3-O-xylosyl-glucoside	[44]
[M + H]^+^	595.5058	6.241	C_27_H_30_O_15_	Skolimoside	[45]
[M − H]^−^	329.3467	4.075	C_17_H_14_O_7_	3,7-Di-O-methylquercetin	[44]
[M + H]^+^	435.4387	6.361	C_20_H_18_O_11_	Quercetin 3-O-xyloside	[44]
[M + H]^+^	433.2267	2.474	C_21_H_36_O_9_	10,11-Epoxy-2,7,8-guaianetriol,2-O-β-D-glucopyranoside	[45]
[M + H]^+^	277.2078	1.6447	C_17_H_24_O_3_	10-Hydroperoxy-1,8-heptadecadiene-4,6-diyn-3-ol	[46]
[M − H]^−^	281.3715	4.023	C_18_H_34_O_2_	6-Octadecenoic acid	[47]

**Table 2:** Biochemical analyses in different studied groups.

	Normal Control	CdCl_2_	CdCl_2_ & Vit. E	CdCl_2_ & RCME
Testosterone (ng/mL)	3.89 ± 0.08	2.84 ± 0.13 ^a^	3.30 ± 0.12 ^a^	3.94 ± 0.2 ^b, c^
LH (ng/mL)	2.47 ± 0.22	1.22 ± 0.13 ^a^	1.81 ± 0.07 ^a, b^	2.28 ± 0.17 ^b^
FSH (ng/mL)	5.52 ± 0.23	8.21 ± 0.25 ^a^	6.15 ± 0.12 ^b^	5.80 ± 0.16 ^b^
Glutathione (mg/g testicular tissues)	4.59 ± 0.30	2.21 ± 0.16 ^a^	2.81 ± 0.07 ^a, b^	2.92 ± 0.06 ^a, b^
Catalase (U/g testicular tissues)	1.69 ± 0.10	1.25 ± 0.08 ^a^	1.53 ± 0.05 ^b^	1.51 ± 0.06 ^b^
Superoxide dismutase (U/g testicular tissues)	1.46 ± 0.06	1.11 ± 0.05 ^a^	1.44 ± 0.07 ^b^	1.44 ± 0.07 ^b^
TAC (mmol/g testicular tissues)	1.87 ± 0.12	1.09 ± 0.08 ^a^	1.85 ± 0.10 ^b^	1.58 ± 0.08 ^b^
MDA (nmol/g testicular tissues)	0.88 ± 0.04	2.12 ± 0.18 ^a^	1.61 ± 0.14 ^a, b^	1.5 ± 0.13 ^a, b^
Nitric oxide (µmol/g testicular tissues)	8.82 ± 0.23	12.26 ± 0.56 ^a^	10.64 ± 0.46	10.77 ± 0.50

Values are means ± SE, with the number of animals = 12 for each group. ^a^ *p* < 0.05 versus normal control group. ^b^ *p* < 0.05 versus CdCl_2_ group. ^c^ *p* < 0.05 versus CdCl_2_ + Vit. E group. Using one-way ANOVA followed by Tukey post-hoc test. RCME, red carrot methanolic extract; LH, luteinizing hormone; FSH, follicle stimulating hormone; TAC, total antioxidant capacity; MDA, malondialdehyde.

## Data Availability

All generated data in this study are included in the article.
